# Co-encapsulation of HNF4α overexpressing UMSCs and human primary hepatocytes ameliorates mouse acute liver failure

**DOI:** 10.1186/s13287-020-01962-7

**Published:** 2020-10-23

**Authors:** Defu Kong, Huiming Xu, Mo Chen, Yeping Yu, Yongbing Qian, Tian Qin, Ying Tong, Qiang Xia, Hualian Hang

**Affiliations:** 1 Department of Liver Surgery, Renji Hospital, School of Medicine, Shanghai Jiao Tong University, 160 Pujian Road, Shanghai, 200127 China; 2 State Key Laboratory of Oncogenes and Related Genes, Renji-MedX Clinical Stem Cell Research Center, Ren Ji Hospital, School of Medicine, Shanghai Jiao Tong University, Shanghai, China

**Keywords:** Acute liver failure, Encapsulation, MSCs, HNF4α, HB-EGF

## Abstract

**Background:**

Acute liver failure (ALF) is a complicated condition that is characterized by global hepatocyte death and often requires immediate liver transplantation. However, this therapy is limited by shortage of donor organs. Mesenchymal stem cells (MSCs) and hepatocytes are two attractive sources of cell-based therapies to treat ALF. The combined transplantation of hepatocytes and MSCs is considered to be more effective for the treatment of ALF than single-cell transplantation. We have previously demonstrated that HNF4α-overexpressing human umbilical cord MSCs (HNF4α-UMSCs) promoted the expression of hepatic-specific genes. In addition, microencapsulation allows exchange of nutrients, forming a protective barrier to the transplanted cells*.* Moreover, encapsulation of hepatocytes improves the viability and synthetic ability of hepatocytes and circumvents immune rejection. This study aimed to investigate the therapeutic effect of microencapsulation of hepatocytes and HNF4α-UMSCs in ALF mice.

**Methods:**

Human hepatocytes and UMSCs were obtained separately from liver and umbilical cord, followed by co-encapsulation and transplantation into mice by intraperitoneal injection. LPS/D-gal was used to induce ALF by intraperitoneal injection 24 h after transplantation. In addition, Raw 264.7 cells (a macrophage cell line) were used to elucidate the effect of HNF4α-UMSCs-hepatocyte microcapsules on polarization of macrophages. The protein chip was used to define the important paracrine factors in the conditioned mediums (CMs) of UMSCs and HNF4α-UMSCs and investigate the possible mechanism of HNF4α-UMSCs for the treatment of ALF in mice.

**Results:**

HNF4α-UMSCs can enhance the function of primary hepatocytes in alginate–poly-L-lysine–alginate (APA) microcapsules. The co-encapsulation of both HNF4α-UMSCs and hepatocytes achieved better therapeutic effects in ALF mice by promoting M2 macrophage polarization and reducing inflammatory response mainly mediated by the paracrine factor HB-EGF secreted by HNF4α-UMSCs.

**Conclusions:**

The present study confirms that the co-encapsulation of HNF4α-UMSC and hepatocytes could exert therapeutic effect on ALF mainly by HB-EGF secreted by HNF4α-UMSCs and provides a novel strategy for the treatment of ALF.

## Background

Orthotopic liver transplantation for patients with acute liver failure (ALF) can improve survival rates as well as quality of life, but this surgery is expensive and complex, limited by shortage of donors [1]. Transplantation of hepatocytes has emerged as a promising candidate to prolong the lifespan of ALF patients waiting to undergo liver transplantation [2]. Nevertheless, the scarcity of donor hepatocytes and the quality of hepatocytes isolated from marginal livers have restrained the development of this technique. Furthermore, primary hepatocytes forfeit their proliferative ability, undergoing phenotypic de-differentiation and metabolic regression after isolation and in vitro culture [3]. Therefore, a feasible approach to improve the quality as well as vitality of human hepatocytes after isolation is urgently needed [4].

Mesenchymal stem cells (MSCs) are characterized by self-renewal, no tumorigenicity, low immunogenicity, and strong immunomodulatory ability, with a great potential in regenerative medicine [5]. Among different types of MSCs, human umbilical cord MSCs (UMSCs), isolated from Wharton’s jelly of umbilical cord, are featured with high proliferation ability and easy availability, having drawn much attention of the researchers [6]. In the field of hepatic disease, applications of UMSCs were reported effective in ameliorating ALF, hepatic ischemia, and reperfusion injury [7, 8]. Furthermore, a recent study revealed that MSCs co-transplanted with hepatocytes is regarded as a more effective treatment for ALF [9]. In such co-transplantation system, MSCs ameliorated the inflammatory reaction and improved the viability and the functioning of both the recipient liver and the donor hepatocytes, while hepatocytes were found to temporarily support the metabolic function in ALF mice [10].

Hepatocyte nuclear factor-4 alpha (HNF4α) is an important transcription factor of the nuclear hormone receptor family and is essential for maintaining a normal liver architecture [11]. Moreover, it also plays a crucial role in hepatic differentiation, metabolic function, and formation of a polarized hepatic epithelium and cell-cell contact [12]. Moreover, as supported by similar research [13, 14], our previous study showed that HNF4α-overexpressing UMSCs (HNF4α-UMSCs) has significantly improved the differential status of hepatocyte-like cells by activating the expression of genes related to hepatocyte function [15].

In order for promoting therapeutic effect of hepatocytes transplantation, more efforts have been made to improve the viability and function of hepatocytes. Of note, microencapsulation permits exchange of nutrients, oxygen, and small molecules, forming *a protective barrier to the transplanted cells.* According to published studies, the microencapsulation of hepatocytes promoted their viability, improving albumin and urea synthesis, also facilitating the circumvention of immune recognition [16, 17]. Combining the profound immune modulation of UMSCs and material advantage of microencapsulation, the present study aimed to investigate the therapeutic potential of co-encapsulation of HNF4α-UMSCs and human primary hepatocytes on LPS/D-gal-induced ALF mice.

## Materials and methods

### Source of human liver specimens

Adult human liver specimens were collected from patients who were undergoing partial hepatectomy or liver transplantation and immediately stored at 4 °C in UW solution (the University of Wisconsin solution, Netherlands) to isolate hepatocytes. This study was approved by the Institutional Ethical Review Committee of Renji Hospital, School of Medicine, Shanghai Jiao Tong University, and all participants gave informed consent for the collection of their liver specimens.

### Cell isolation and culture

Isolation of human hepatocytes was performed as described previously [18]. Briefly, the liver tissue was perfused through intrahepatic vein with PBS for 15 min at 37 °C and then digested with collagenase IV (Sigma, MO, USA) for 25 min. Next, mechanical destruction and filtration of the liver tissue were performed through a 70-μm cell strainer to obtain hepatocytes suspension. Finally, the cell pellet was washed twice using GBSS (Gibco, MA, USA) and centrifuged at 80×*g* for 10 min. The isolated hepatocytes were cultured in Williams’ Medium E (Gibco, MA, USA) with 10% fetal bovine serum (FBS, Gibco, MA, USA).

The primary mice hepatocytes were isolated and cultured as previously described [19]. UMSCs were cultured in DMEM/F12 supplemented with 10% FBS. UMSCs between passage 3 (P3) and P5 were used in the following experiments.

### Encapsulation of cells in alginate–poly-L-lysine–alginate microcapsules

Hepatocytes and UMSCs were immobilized into alginate–poly-L-lysine–alginate (APA) microcapsules by syringe-extrusion technique as previously reported. Briefly, the hepatocytes and the UMSCs were suspended in 0.9% sodium chloride containing 1.5% alginate (Sigma, MO, USA) at a density of 2–5 × 10^6^ cells/ml. The cell suspension was forced out through an encapsulator (NISCO, Zurich, Switzerland), and the beads were allowed to gel in a hardening bath buffer containing 100 mmol/L of CaCl_2_ (Sigma-Aldrich, MO, USA) and 10 mmol/L of MOPS (Sigma-Aldrich, MO, USA). After 10 min of hardening, the beads were washed thrice with PBS, and the microspheres were coated with 0.05% (w/v) poly-L-lysine for 10 min, followed by washing with the buffer containing 0.1% CHES, 1.1% CaCl_2_ and 0.9% NaCl. Furthermore, the microspheres were exposed to 0.15% sodium alginate for 4 min to form the outer layer of the membrane, and then the droplets were washed with saline. The diameter of the microcapsules was approximately 250–350 μm.

### Measurement of albumin and urea production

Microcapsules of human hepatocytes (1 × 10^5^ cells) alone or with UMSCs at a ratio of 10:1 or 5:1 or 2.5:1 were seeded in a 24-well plate. The supernatant was collected from day 2 to 10. The concentration of albumin and urea was measured using albumin ELISA kit (Bethyl Laboratories, TX, USA) and a urea assay kit (Bioassay System, CA, USA) following the manufacturer’s instructions.

### Experimental design and animal groups

Male 8–10 weeks old C57BL/6 mice were purchased from Shanghai SLAC Co. Ltd. (Shanghai, China). All animal experiments were approved by the Institutional Animal Care and Use Committee of the Shanghai Jiao Tong University School of Medicine (No. SYKX-2008-0050). For intraperitoneal (i.p.) transplantation of microcapsules, the mice were randomly divided into four groups with eight mice per group. The groups were as follows: SHAM (blank control, without administration of LPS and D-gal), CON (control, APA), HEP (hepatocytes, 5 × 10^6^), UMSC-HEP (UMSCs 2 × 10^6^ and hepatocytes 5 × 10^6^ cells), and HNF4α-UMSC-HEP (HNF4α-UMSC 2 × 10^6^ and hepatocytes5 × 10^6^ cells). ALF was induced by i.p. injection of the combination of LPS (50 μg/kg) and D-gal (800 mg/kg) dissolved in PBS for 24 h after microcapsule transplantation. The HB-EGF-neutralizing antibody (anti-HB-EGF, R&D, MN, USA) and control IgG (AB-108-C, R&D, MN, USA) were reconstituted in sterile PBS and administered to ALF mice (10 μg/injection, i.p.).

The enzyme activities of aspartate aminotransferase (AST) and alanine transaminase (ALT) were measured using a standard clinical automatic analyzer (Dimension Xpand; Siemens Dade Behring, Munich, Germany). The levels of TNF-α, CXCL15 (a homolog of human IL-8), and LDH were detected by ELISA kits (Nanjing Jiancheng Bioengineering Institute, China) according to the manufacturer’s instructions.

### Histopathology, immunohistochemistry, and immunofluorescence analyses

Liver tissues were fixed in 10% formalin and embedded in paraffin, and then were cut into 5-μm thick sections. The liver sections were then stained with hematoxylin and eosin (HE) for histopathological examination. The histopathological changes were given scores according to a classic liver injury score standard [19]. Immunohistochemistry analyses were performed as previously described [20]. For immunohistochemistry, the liver sections were incubated with primary antibodies against MPO (Abcam, Cambridge, UK) or F4/80 (Abcam). After washing with PBS, the sections were incubated with the biotinylated secondary antibody anti-rabbit IgG (Vector, Peterborough, UK). Then, the sections were visualized using a Vectastain ABC Kit (Vector) at RT 30 min and developed with 3,3′-diaminobenzidine (DAB) at RT. The imagines were visualized by using an inverted microscope. Manual counts were performed to estimate the number of MPO or F4/80 positive cells in the liver of mice in different groups.

For immunofluorescence analyses, the liver sections were incubated with the following primary antibodies: anti-CD68, anti-CD86 and anti-CD206 (all from Abcam, Cambridge, MA, USA). After washing in PBS, the tissue sections were incubated with corresponding conjugated secondary antibodies. The images were then visualized by using an inverted fluorescence microscope. The quantification results were evaluated in at least six representative visual fields for each group in a blinded manner by an experienced pathologist. Image-Pro Plus 6.0 (Media cybernetics, Silver Springs, MD, USA) was used for image analysis.

### Protein chip

Protein antibody array was performed to test the secretion factors in the conditioned medium (CM) of HNF4α-UMSCs and UMSCs. The expression levels of 1070 human target proteins in cell supernatant were detected. The procedure was done according to the manual of the manufacturer (Aksomics, Shanghai, China). In brief, the protein concentration of the CM was firstly tested by BCA Protein Assay Kit (Kang Chen Corporation, KC-430, China), followed by adding the CM onto the blocked protein array membranes, and then incubating at room temperature (RT) for 2 h. Next, the membranes were washed and incubated with biotin-conjugated antibodies at RT for 2 h and then reacted with HRP-conjugated streptavidin at RT for 2 h. Finally, the membranes were exposed to X-ray films and developed using a film scanner. The intensity of the signals was quantified by densitometry.

### Dual-luciferase reporter assay

The cells were cultured into 96-well plates, and were transfected by Lipofectamine TM 3000 (Invitrogen, CA, USA) with the plasmids of HB-EGF promoters, pWPXL, or HNF4α, and internal control PRL-TK reporter plasmid after 24 h. After culturing for 48 h, the luciferase activities of the cells were measured by dual-luciferase reporter assay kit (Promega, WI, USA). The experiment was repeated at least three times.

### Flow cytometry analysis and cell sorting

Mice were sacrificed and the livers were minced and tamped by using a 70-μm filter, the liver leukocytes were purified with 35% Percoll gradient (GE Healthcare), and then the red blood cells were lysed with RBC lysis buffer (ebioscience, MA, USA). The leukocytes were stained with fixable viability dye (ebioscience) and then incubated with Fc block (BD Biosciences), followed by staining with the antibodies against F4/80 (mouse PE T45-2342), CD11b (mouse FITC M1/70), and CD206 (mouse CD206 Alexa 647 MR5D3) (all from Biolegend, CA, USA). Finally, flow cytometry was performed by Cyto FLEX flow cytometer (Beckman coulter, Fullerton, CA, USA) and analyzed with Flowjo software (Treestar, OR, USA).

### Statistical analysis

Data were presented as means ± SEM (*n* ≥ 3 experiments), and statistical significance were determined using Student’s *t* test. *P < 0.05, **P < 0.01, ***P < 0.005, and **** *P* < 0.001; ^#^*P* < 0.05 and ^##^*P* < 0.01.

## Results

### HNF4α-UMSCs enhances the function of human primary hepatocytes in APA microcapsules

**Fig. 1:**
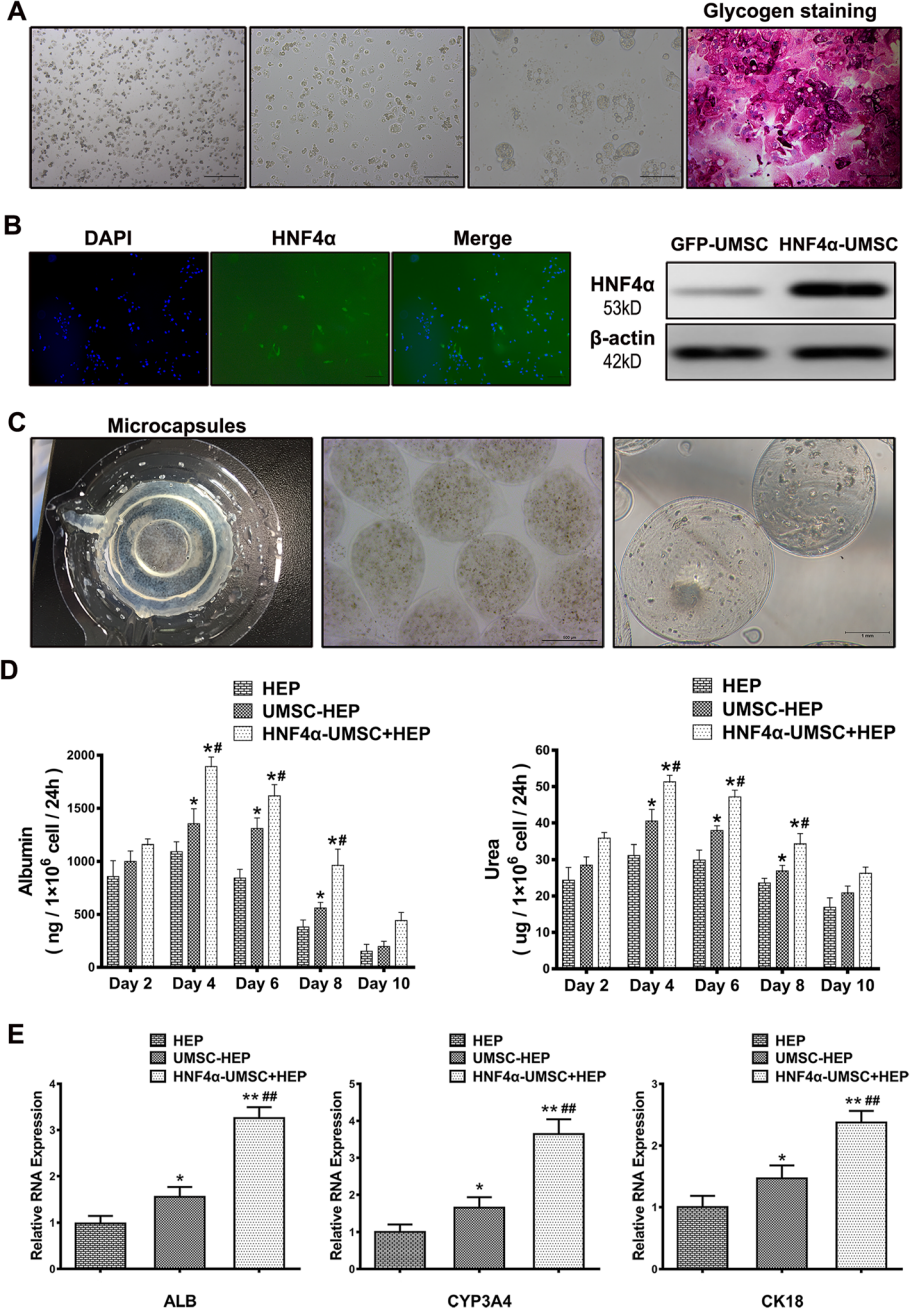
HNF4α-UMSCs enhance the function of human primary hepatocytes in APA microcapsules. **a** Phase contrast of human primary hepatocytes and image of glycogen staining. **b** Overexpression of HNF4α in UMSCs was confirmed by fluorescence and western blotting against HNF4α antibody. **c** Phase contrast of microcapsules of primary hepatocytes with/without HNF4α-UMSCs or UMSCs. **d** Measurement of the levels of albumin and urea in the supernatant of microcapsules in the groups of HEP, UMSC-HEP, and HNF4α-UMSC-HEP at various time points. **e** The mRNA expression levels of hepatocyte-specific genes, ALB, CYP3A4, and CK18 by qPCR analysis. Total RNA was extracted on day 4. The levels of mRNA in HEP was set as 1. Data were collected from at least six separate experiments and are presented as means ± S.E.M. Statistical significance was tested by Student’s *t* test. *P < 0.05 and ** *P* < 0.01 compared with CON. ^#^*P* < 0.05 and ^##^*P* < 0.01 compared with UMSC-HEP

The trophic factors secreted by MSCs were found to be efficient in improving the hepatocyte function in ALF [21]. To assess whether HNF4α-UMSCs could promote better function of human hepatocytes in the microencapsulation system, we initially isolated human hepatocytes from liver specimens and confirmed the phenotype of hepatocytes by glycogen staining (Fig. 1a). Overexpression of HNF4α in UMSCs was then validated by western blotting and fluorescence (Fig. 1b). To explore the optimal co-culture ratio of the microencapsulation system, hepatocytes and HNF4α-UMSCs were encapsulated sequentially at ratio of 10:1, 5:1, and 2.5:1, followed by culturing for 10 days. The albumin and the urea levels of hepatocytes were detected by ELISA kits from day 2 to 10, as essential functional biomarkers to evaluate function state of hepatocytes. As shown in Fig. S1, when hepatocytes and HNF4α-UMSCs were encapsulated at the ratio of 2.5:1, the levels of albumin and urea reached to the peak, suggesting the maximum of hepatocytes functions. Therefore, the co-culture ratio of 2.5:1 was set for following experiments. In order for further exploring the optimal timespan for the microencapsulation system, co-culturing system including hepatocytes with UMSCs (UMSC-HEP) and hepatocytes with HNF4α-UMSCs (HNF4α-UMSC-HEP) at a ratio of 2.5:1 were encapsulated (Fig. 1c). Next, the microcapsules were cultured for 10 days and then the supernatant was harvested every 2 days for albumin and urea assay, while human hepatocytes alone (HEP) serves as negative control. The results revealed that albumin secretion and urea production increased gradually to the peak on day 4, and then gradually declined from day 6 to 10 (Fig. 1d). In addition, UMSCs promoted albumin secretion and urea production of hepatocytes, while the HNF4α-UMSC more efficiently enhanced the protective effects on hepatocytes than UMSCs. To further confirm whether HNF4α-UMSCs enhance the function of hepatocytes via secretions, the hepatocytes and different HNF4α-UMSCs were co-cultured in two layers of transwell-chambers for 4 days. Hepatocytes in the lower layer were then harvested. Real-time PCR was performed to examine the expression levels of hepatocyte genes like Albumin (ALB), CYP3A4, and CK18 in different groups presenting as liver specific markers for hepatic maturation and liver functions. As shown in Fig. 1e, the expression levels of ALB, CYP3A4, and CK18 were higher in HNF4α-UMSC-HEP group than those in the HEP or UMSC-HEP group, suggesting that the HNF4α-UMSCs exerted the strongest promotion on hepatocytes via secreting certain effective factors. Taken together, these results indicated that HNF4α-UMSCs in APA microcapsules could significantly improve the viability and function of primary hepatocytes.

### Co-encapsulation of HNF4α-UMSCs and human hepatocytes ameliorated liver injury in ALF mice

**Fig. 2:**
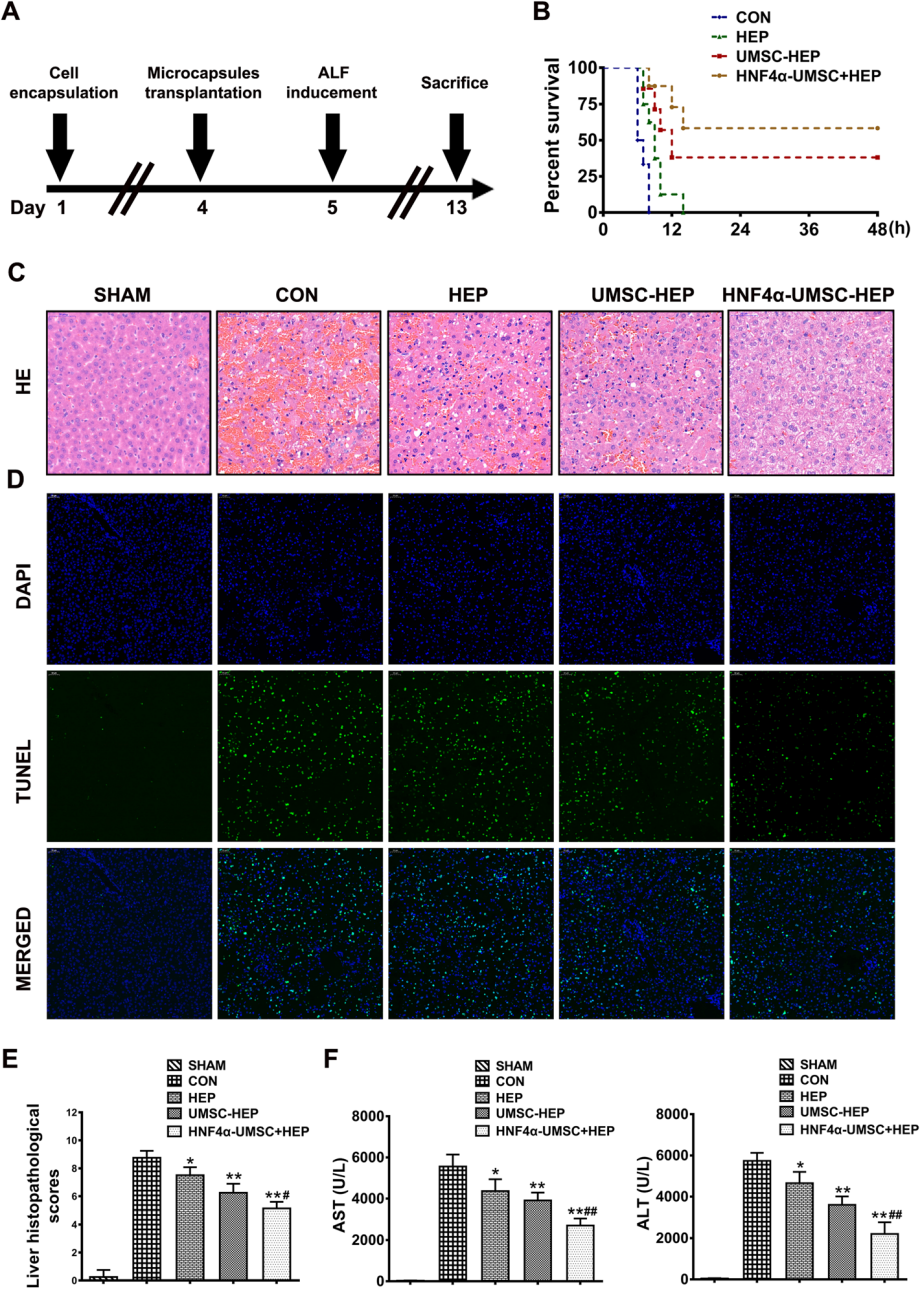
Co-encapsulation of HNF4α-UMSCs and human hepatocytes ameliorated ALF in mice. **a** Schematic representation of experimental procedure design. Mice were transplanted with control or HEP, UMSC-HEP, and HNF4α-UMSC-HEP 24 h before LPS/D-gal challenge. **b** Survival rate of mice injected with LPS/D-gal in Control (CON), HEP, UMSC-HEP, and HNF4α-UMSC-HEP groups (*n* = 6–12). **c** Liver tissues were harvested 6 h after LPS/D-gal challenge for histopathological examination using H&E staining (Original magnification × 200). Representative images are displayed. **d** Immunofluorescence staining of liver sections of ALF mice with antibodies against TUNEL with DEPI. **e** Liver histopathological scores. Data were collected from at least six separate fields of different mice. **f** The serum ALT and AST activities were measured 6 h after intraperitoneal injection of LPS/D-gal (*n* = 6). Data were collected from at least six separate experiments and are presented as means ± S.E.M. *P < 0.05 and **P < 0.01 compared with CON. ^#^*P* < 0.05 and ^##^*P* < 0.01 compared with UMSC-HEP

To assess whether HNF4α-UMSC-HEP have a protective role in ALF mice model, the microcapsules were cultured for 4 days based on above data (Fig. 1d, e). Then, microcapsules containing different cellular compositions [HNF4α-UMSC-HEP, UMSC-HEP, HEP, or simple APA alone (labeled as Sham)] were transplanted into mice by intraperitoneal injection (i.p.). Twenty-four hours later, LPS (50 μg/kg) and D-gal (800 mg/kg) were used to induce ALF by i.p. (Fig. 2a). During the 7-day follow-up period, after transplantation of the microcapsules, all mice in the control group (CON) progressed to death, with a mortality rate of 100%. In contrast, only three mice died in the HNF4α-UMSC-HEP group during the observation, indicating a better survival rate of mice than other groups (Fig. 2b). As depicted in Fig. 2c, there was an evident increase of the hepatic necrotic areas in the mice of CON group after 6 h of injection of LPS and D-gal. Interestingly, a reduced hepatic necrotic level was observed in the other groups. Moreover, HNF4α-UMSC-HEP showed the least hepatic necrotic areas compared to HEP or UMSC-HEP. Immunofluorescence staining of TUNEL (an apoptosis marker) indicated a remarkable reduction of hepatic apoptosis in the group of UMSC-HEP and HNF4α-UMSC-HEP (Fig. 2d). Then, the HNF4α-UMSC-HEP pretreated mice demonstrated a more significant improvement of the architecture of the liver and were characterized by decreased levels of edema, necrosis, and neutrophil infiltration when compared to those in the mice of other groups (Fig. 2c, e) [22]. Consistent with these histological alterations, the levels of serum AST and ALT in the serum o mice also considerably decreased in the HNF4α-UMSC-HEP pre-treatment (Fig. 2f). These results demonstrated that HNF4α-UMSC-HEP significantly attenuated the liver injury in ALF mice.

### Co-encapsulation of HNF4α-UMSCs and human hepatocytes alleviated inflammatory responses in ALF mice

**Fig. 3:**
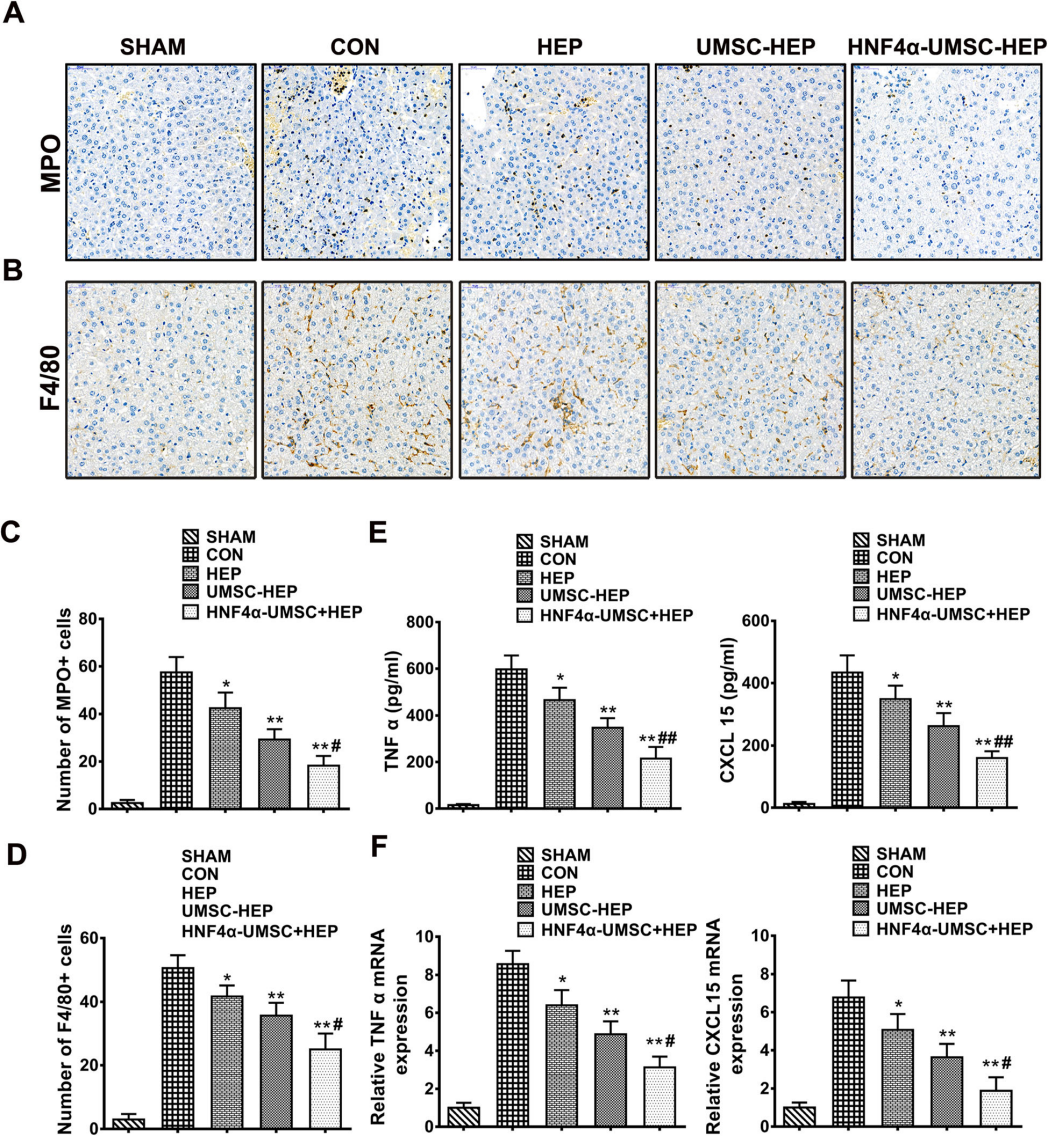
Co-encapsulation of HNF4α-UMSCs and human hepatocytes alleviated inflammatory responses in ALF mice. **a** Immunochemistry images of liver sections with MPO antibodies (original magnification, × 200). **b** Immunochemistry images of liver sections with F4/80 antibodies (original magnification, × 200). **c** Quantification of MPO positive cells in livers. **d** Quantification of F4/80 positive cells in livers. **e** ELISA analysis of TNF-α and CXCL15. **f** The mRNA levels of TNF-α and CXCL15 levels in the serum of ALF mice by real-time PCR analysis. Data were collected from at least six separate experiments and are presented as means ± S.E.M. *P < 0.05 and **P < 0.01 compared with CON. ^#^*P* < 0.05 and ^##^*P* < 0.01 compared with UMSC-HEP

Acute inflammatory response has been widely identified as a detrimental hallmark of ALF, thus enhancing inflammation resolution can greatly protect liver from injury by inhibiting acute inflammation [23]. Here, we wondered whether the attenuated liver injury in HNF4α-UMSC-HEP group was attributed to the enhanced inflammation resolution effect. As shown in Fig. 3a and c, fewer myeloperoxidase (MPO, a neutrophil marker) positive cells were observed in the mice of HNF4α-UMSC-HEP group compared to those in the other groups, indicating a decreased neutrophil infiltration. Similarly, fewer F4/80 (known as a marker for macrophages) positive cells were also observed in the group of HNF4α-UMSC-HEP (Fig. 3b, d). The levels of proinflammatory cytokines such as TNF-α and CXCL15 in the serum of mice decreased in HEP, UMSC-HEP, and HNF4α-UMSC-HEP groups compared to those of CON group, while the lowest levels of TNF-α and CXCL15 were shown in the group of HNF4α-UMSC-HEP compared to those of other groups (Fig. 3e). Furthermore, mRNA levels of TNF-α and CXCL15 of the livers of mice showed that HNF4α-UMSC-HEP exerted a similar inhibitory effect on the proinflammatory cytokines (Fig. 3f). These data indicated that HNF4α-UMSC-HEP relieved liver injury induced by LPS/D-gal by alleviating the inflammatory responses in mice.

### Co-encapsulation of HNF4α-UMSCs and human hepatocytes promoted M2 polarization of macrophages both in vitro and in vivo

**Fig. 4:**
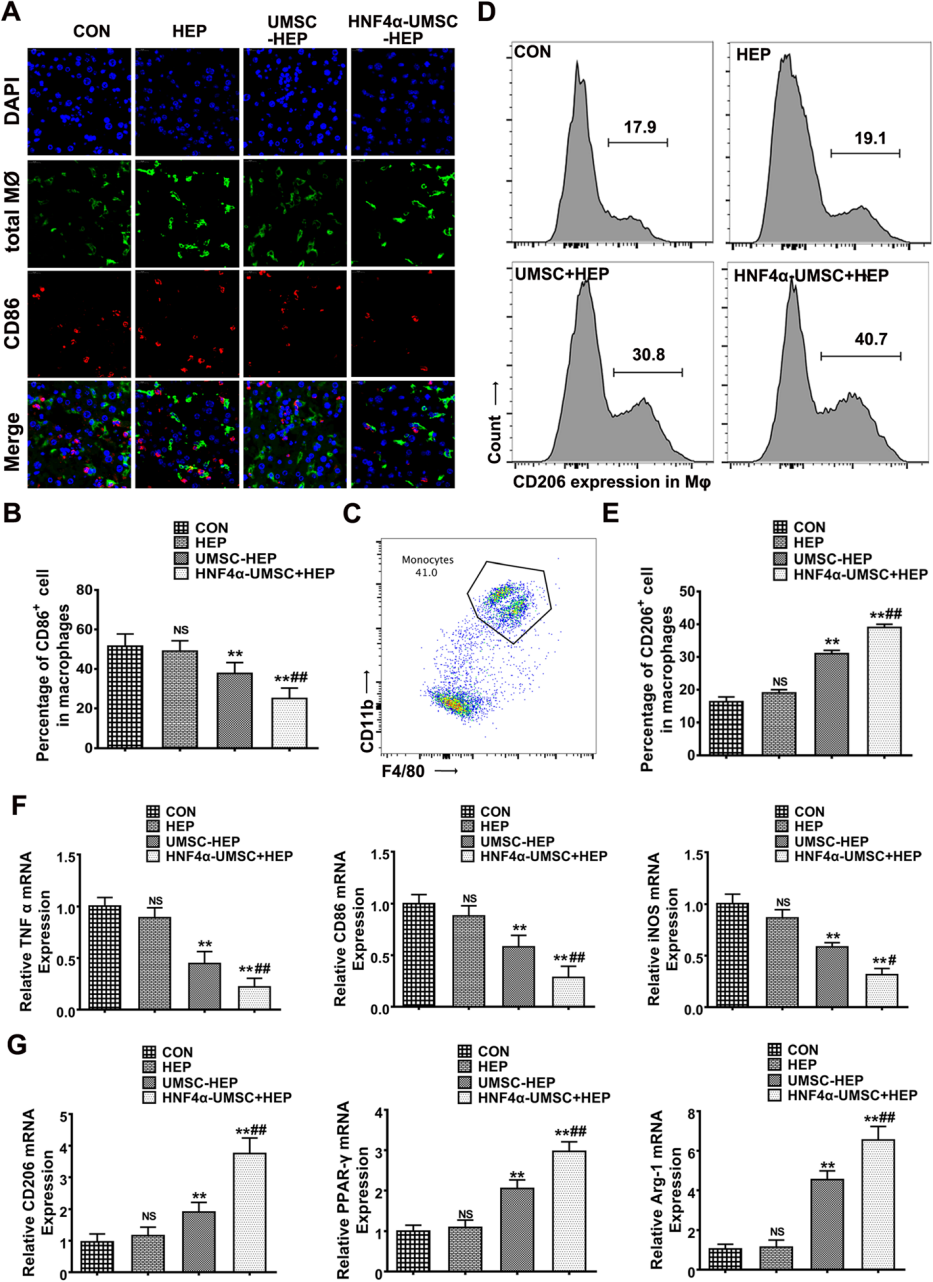
Co-encapsulation of HNF4α-UMSCs and human hepatocytes promoted M2 polarization of macrophages. **a** Immunofluorescence staining of liver sections of ALF mice with antibodies against CD68 and CD86. **b** Percentage of CD68^+^ cells in macrophages under a microscopic field. Eight sections were analyzed for each liver and counted each section at least in four fields. **c** Gate strategy of flow cytometry analysis. **d** Flow cytometry analysis of CD206^+^ cells in macrophages. **e** Percentage of CD206^+^ cells in macrophages (Mφ) (*n* = 4–6). **f** The mRNA expression levels of TNF-α, CD86, iNOS, CD206, Arg-1 and PPAR-γ in Raw264.7 cells treated with CM of different groups after challenged with LPS. Data are expressed as means ± S.E.M. (*n* = 6). *P < 0.05 and **P < 0.01 compared with CON, ^#^*P* < 0.05 and ^##^*P* < 0.01 compared with UMSC-HEP

Increasing evidence demonstrates that the phenotypic change of macrophages from activated M1 state to suppressor M2 state indicates a change from proinflammatory stage to anti-inflammatory stage. LPS is known to induce M1 polarization of macrophages, as a novel mechanism inducing sequential inflammatory response of ALF [24–26]. To determine whether the therapeutic effect of HNF4α-UMSC-HEP on ALF was related to the M1 polarization of macrophages induced by LPS/D-gal, immunostaining analysis of liver tissues with the antibodies against CD68 (a total macrophage marker) and CD86 (M1 macrophage marker) was performed. As shown in Fig. 4a and b, higher percentages of CD86 positive cells over the total CD68 positive cells were observed in the liver of the mice in the control group and HEP group. However, there was a significant reduction in the percentages of CD86 positive cells in the UMSC-HEP group and HNF4α-UMSC-HEP group compared to those in the CON and HEP groups. Moreover, the lowest percentage of CD86 positive cells was observed in the HNF4α-UMSC-HEP group compared to the other groups (Fig. 4a, b). The data revealed that HNF4α-UMSCs significantly reduced the ratio of M1 macrophages. On the other hand, to further determine the suppressive effects of UMSC-HEP and HNF4α-UMSC-HEP on inflammatory response, flow cytometry of liver lymphocytes of mice in all groups was performed. As shown in Fig. 4c and d, UMSC-HEP and HNF4α-UMSC-HEP evidently increased the percentages of CD206 (M1 macrophage marker) positive cells over the total CD68 positive cells. Moreover, a profound increase of CD206 positive cells was observed in the HNF4α-UMSC-HEP compared to UMSC-HEP. These results suggested that HNF4α-UMSC-HEP could efficiently promote M2 polarization of macrophages, protecting the liver from LPS/D-gal-induced injury. To further elucidate the effect of HNF4α-UMSC-HEP on polarization of macrophages, LPS was used to induce the murine macrophage cell line Raw264.7 cells to polarize towards M1 phenotype. Then, the cells were separately co-cultured with conditioned medium (CM) of CON, HEP, UMSC-HEP, and HNF4α-UMSC-HEP. As shown in Fig. 4e, the mRNA levels of TNF-α, CD86 and inducible nitric oxide synthase (iNOS) (M1 macrophage markers) were evidently higher in the CON group and HEP group after exposure to LPS, conversely, the mRNA levels of M2-macrophage markers, such as CD206, peroxisome proliferation-activated receptor-gamma (PPAR-γ), and Arginase-1 (Arg-1) were obviously lower in the control group and HEP group (Fig. 4f). However, the mRNA levels of M1 macrophage markers were significantly reduced when the cells co-cultured with the CM of UMSC-HEP or HNF4α-UMSC-HEP. Moreover, the CM of HNF4α-UMSC-HEP exerted a stronger capacity to downregulate the expression of M1 macrophage markers and upregulate the expression levels of M2-macrophage markers than those in the other groups (Fig. 4f). Taken together, these results suggested that HNF4α-UMSC-HEP could more efficiently promote polarization of macrophages towards M2 type both in vivo and in intro*.*

### Paracrine factors of HNF4α-UMSCs contribute to its protective effects on injured hepatocytes induced by LPS/D-gal

**Fig. 5:**
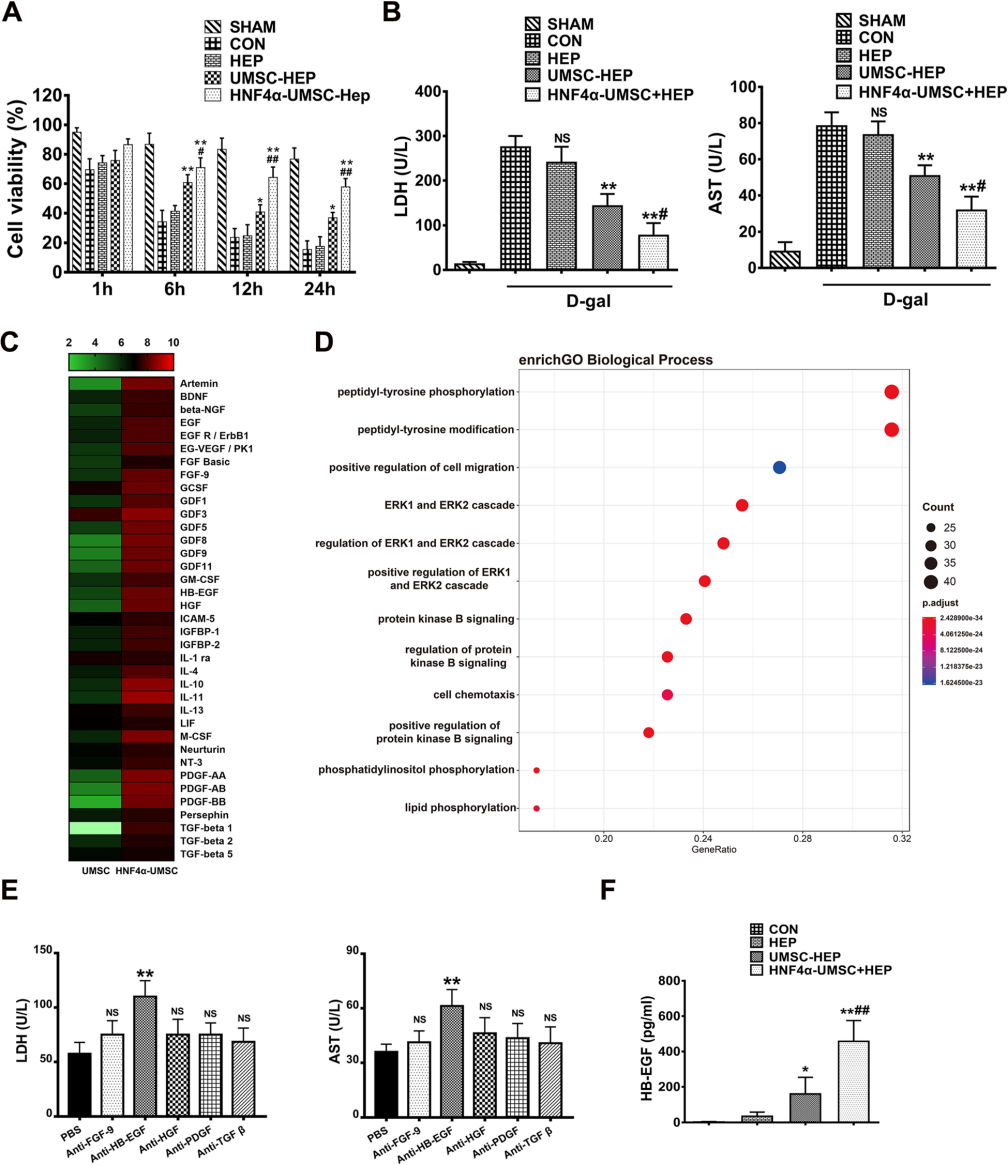
Conditioned medium of HNF4α-UMSCs prevented hepatocytes from D-gal-induced injury. **a** Analysis of viability of mouse primary hepatocytes in different groups at different time points by CCK-8. **b** Analysis of LDH and AST levels in different groups. **c** The relative concentrations of proteins of the condition mediums of UMSCs and HNF4α-UMSCs that obtained a significant score (*q*-value < 0.001%) are presented in a “heatmap”. Low concentration is shown in green, medium concentration is shown in black and high concentration is shown in red. **d** GO enrichment analysis of biological processes of high-level proteins in CM of HNF4α-UMSCs. Normalized array data of proteins were analyzed by SAM to detect differences in the concentrations between HNF4α-UMSCs-CM and UMSCs-CM. **e** The levels of LDH and AST in the medium of hepatocytes injured by D-gal with CM of HNF4α-UMSCs-CM, and neutralizing antibodies against different proteins. **f** ELISA analysis on HB-EGF concentration in the CM of UMSC and HNF4α-UMSC. Data are expressed as means ± S.E.M. (n = 6). *P < 0.05 and **P < 0.01 compared with CON, ^#^*P* < 0.05 and ^##^*P* < 0.01 compared with UMSC-HEP

To further illustrate the protective effects of HNF4α-UMSCs on liver injury, we applied D-gal (10 mg/mL) to induce the injury of primary mouse hepatocytes and simultaneously co-cultured the injured cells with the CM of cells in the different groups. The cell viability was tested at 1 h, 6 h, 12 h, and 24 h after the treatment with D-gal. The results indicated that the CM of UMSC-HEP and HNF4α-UMSCs-HEP significantly reduced the injury of hepatocytes induced by D-gal, especially for the CM of HNF4α-UMSCs-HEP (Fig. 5a). To further measure injury severity of hepatocytes, the levels of LDH and AST in the supernatant of the cells after treatment with D-gal for 12 h were tested. As shown in Fig. 5b, low levels of LDH and AST in UMSC-HEP group and HNF4α-UMSC-HEP group were observed especially for HNF4α-UMSC-HEP. The above data revealed that the CM of HNF4α-UMSC more efficiently reduced the injury of hepatocytes. To further illustrate what secretory factors contributed to protecting the injured hepatocytes, a protein array of 1080 factors was performed with the CMs of UMSCs and HNF4α-UMSCs, respectively, and relative expression levels of 1080 soluble proteins could be simultaneously detected. Next, we mainly focused on the neurotrophic factors, growth factors, cell adhesion molecules and anti-inflammatory factors which have been implicated previously involved in the repair of liver injury [8, 27–29]. The heatmap shows that the relative expression levels of these secretory factors in the HNF4α-UMSCs were higher than those of UMSCs (Fig. 5c, Table S2). Growth factor FGF9, HB-EGF, HGF, PDGF and TGF-β, anti-inflammatory factors IL-1ra, IL-10, and IL-11, were in the CM of HNF4α-UMSCs than the CM of UMSCs. Furthermore, Gene Ontology (GO) function enrichment analysis of biological process (BP) revealed that ERK and AKT (protein kinase B) signaling pathways were activated in the CM of HNF4α-UMSCs (Fig. 5d), which are important signaling pathways to cell proliferation and migration [30], improving anti-apoptotic effects and attenuating liver injury [31]. To further explore which secreted factor mainly play essential role on injured hepatocytes, antibody neutralizing experiments using blocking antibodies against IgG, FGF9, HB-EGF, HGF, PDGF, and TGF-β on injured hepatocytes were performed. The primary mouse hepatocytes were injured by D-gal and co-cultured with the CM of HNF4α-UMSC-HEP containing a corresponding blocking antibody for 12 h. The levels of LDH and AST in the supernatant were then measured. As shown in Fig. 5e, the levels of LDH and AST were significantly increased in the presence of neutralizing antibody against HB-EGF, while no significant effect was observed in the groups with other neutralizing antibodies. To validate the main source of secreted HB-EGF, we then detected the concentrations of HB-EGF in the CM of HNF4α-UMSCs and UMSCs separately via EILSA (Fig. 5f). CM of HNF4α-UMSCs contained higher concentration of HB-EGF compare to its counterpart, indicating HNF4α-UMSCs as the main source of HB-EGF. These results indicated that HB-EGF secreted by HNF4α-UMSC-HEP might exert a protective effect on the ALF mice.

### Activation of HB-EGF by HNF4α mediated the protection of HNF4α-UMSC-HEP on ALF mice

**Fig. 6:**
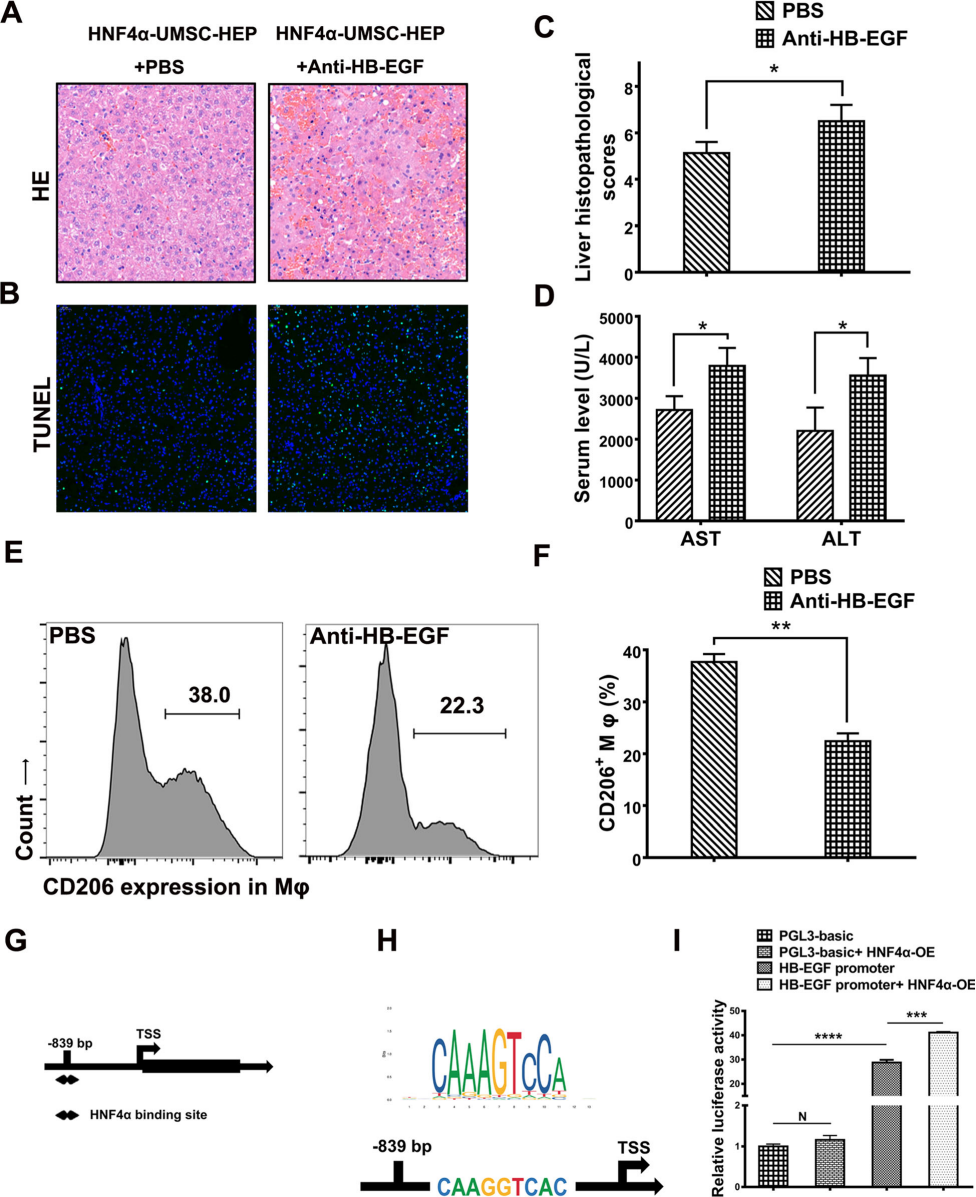
HB-EGF induced by HNF4α accounted for HNF4α-UMSC-HEP-meditated protection. Mice in HNF4α-UMSC-HEP group were treated with LPS/D-gal and with or without neutralizing HB-EGF antibody. **a** Liver samples were harvested 6 h after LPS/D-gal challenge for H&E staining, and the representative images are displayed. **b** Immunofluorescence staining of liver sections of ALF mice with antibodies against TUNEL. **c** The liver histopathological scores were collected from the data of at least six separate experiments. **d** The serum ALT and AST activities were measured in HNF4α-UMSC-HEP group with or without neutralization of HB-EGF antibodies. **e**, **f** Representative FACS plots and statistical quantification of CD206^+^ macrophages upon anti-HB-EGF in HNF4α-UMSC-HEP group (*n* = 4–6). **g** HNF4α-targeted sites on HB-EGF promoter were predicted by the JASPAR website. **h** The sequence of HNF4α potential binding site on HB-EGF promoter by JASPAR (upper panel) and a diagram of HNF4α predicted the binding site (lower panel). **i** The relative luciferase activities of HB-EGF promoter with or without HNF4α expression vectors upon transfection in 293T cells (NC stands for the control of HNF4α plasmid, and PGL3-basic stands for the control of plasmid of HB-EGF promoter). Data are presented as means ± S.D. (*n* ≥ 3). *P < 0.05, **P < 0.01, ***P < 0.005, and ****P < 0.001

To evaluate the therapeutic potentiality of HB-EGF in vivo*,* HB-EGF neutralization antibody was intraperitoneally injected into the ALF mice. Six hours later, the liver injury was assessed. As shown in Fig. 6a–d, the enhanced LPS/D-gal-induced liver injury was observed in the present of neutralizing antibody against HB-EG, as evidenced by increased hepatic necrosis, hepatic apoptosis (Fig. 6a–c), and ALT/AST levels (Fig. 6d). To further assess the essential influence of HB-EGF on inflammation resolution effect on ALF mice, we established HB-EGF knockdown phenotype in HNF4α-UMSCs (labeled as HNF4α-HF^KD^-UMSC). The knockdown effectiveness was confirmed by qRT-PCR and Western blotting (Fig. S2A). Then, HNF4α-HF^KD^-UMSC was co-transplanted into ALF mice with hepatocytes. As contrasted to co-transplantation of HNF4α-UMSCs and hepatocytes, knockdown of HB-EGF significantly attenuated the therapeutic and anti-inflammatory effect of the co-encapsulation system, as suggested by HE sections of liver tissue, as well as immunohistochemistry against F4/80 and MPO. We found more severe injured the liver was, more infiltrated neutrophil (suggested by MPO) and macrophages (suggested by F4/80) when HB-EGF was knocked down (Fig. S2B). Similarly, mRNA levels of inflammatory cytokines like TNF-α and CXCL15 in liver tissue also elevated when HB-EGF was knocked down in in HNF4α-UMSCs, so did the levels of ALT and AST (Fig. S2C). In addition, HB-EGF is demonstrated to play a critical role in the polarization of macrophages [32]. To explore whether HB-EGF affects the polarization of macrophages in the livers of ALF mice, flow cytometry was performed. As shown in Fig. 6e and f, the percentage of M2 macrophages over total macrophages was obviously decreased in the present of neutralizing antibody against HB-EG, indicating that HB-EGF exerts an important role on the relief of liver injury and the promotion of macrophages M2 polarization.

On the other hand, HNF4α is regarded as an important transcription factor that regulates genes expression in hepatocytes as well as others cells [33]. To explore the mechanism of HNF4α on the expression of HB-EGF, the HB-EGF promoter region was analyzed by JASPAR database (http://jaspar.genereg.net/) and the potential binding sequence of HNF4α was predicted, which is located at about − 839 to about − 848 bp relative to the promoter site (Fig. 6g, h). To verify the effects of HNF4α on HB-EGF expression, the plasmids of HNF4α and HB-EGF promoter luciferase reporter (HB-EGF promoter) of 4848 bp with HB-EGF promoter region was transfected in HEK 293 cells and PGL3-basci was used as a control of HB-EGF promoter. As shown in Fig. 6i, HNF4α significantly activated the expression of HB-EGF reporter. As a control, no effect on PGL3 basic was observed in the HNF4α group without HB-EGF promoter. These results indicate that HNF4α binds to HB-EGF promoter and activates the expression of HB-EGF. In general, HB-EGF secreted by HNF4α-UMSC contributed to the protective effects of encapsulation of HNF4α-UMSC-HEP on ALF mice.

## Discussion

Cell-based therapies have emerged as potential alternatives to liver transplantation for the treatment of ALF due to their feasibility and low-invasive nature. Increasing evidence demonstrates that mesenchymal stem cells (MSC) and hepatocytes have a benefit therapeutic effect on ALF. However, the survival and viability of transplanted cells directly affect their therapeutic effects [34]. Immune rejection is considered an important impact on the effects. Nowadays, microcapsules of cells are a good strategy to prevent immune injection [35]. The microcapsules are used to construct an immuno-isolation membrane, which is tolerant from immune recognition and immune attack. Microcapsules have a porous structure that allows oxygen and other nutrients to nourish the encapsulated cells. Moreover, they also provide a diffusive control of surrounding vasculature or tissue by the paracrine factors secreted by the encapsulated cells. Previous studies showed that MSCs in the microcapsules with porcine hepatocytes enhanced the viability and function of porcine hepatocytes [36, 37]. Therefore, we used APA to microcapsule UMSCs with human primary hepatocytes and transplanted these microcapsules into ALF mice to observe their effects on ALF in the present study (Figs. 1c and 2a). The results showed that transplantation of co-microcapsule of UMSCs and hepatocytes significantly attenuated liver injury, resulting in improved survival rate of LPS/D-gal-induced ALF mice (Fig. 2b–e). Moreover, hepatocytes co-transplanted with UMSCs in APA microcapsule can compensate the function of injured liver, preserving as substitute cellular source of large scale of quantities for liver function.

Previous studies have demonstrated that co-transplantation of hepatocytes and MSCs exerted a significant therapeutic effect on ALF animal models, in which MSCs support hepatocellular metabolism and stabilization in ALF [38]. In the current study, in the co-capsulation system of UMSCs and hepatocytes, in vitro, UMSCs promoted the synthesis and secretion of human primary hepatocytes and enhanced the viability and function of hepatocytes (Fig. 1d, e). In vivo, UMSC-HEP-APA reduced decreased mortality rate, hepatic apoptosis and necrotic level (Fig. 2), inhibited inflammatory response including an alleviated neutrophil and macrophage infiltration in the liver, inflammatory factors levels (Fig. 3). In addition, polarization of macrophages was also involved in the inflammatory responses of ALF [39, 40]. The phenotypic change of macrophages from activated M1 state to suppressor M2 state indicates a change from proinflammatory stage to anti-inflammatory stage [41]. We herein found the dramatically increased portion of M1 macrophages in the livers of LPS/D-gal-induced ALF mice. Moreover, the number of M1 macrophages was reduced and the number of M2 macrophages was increased in the livers of ALF mice in UMSC-HEP group (Fig. 4a–d). The CM of HNF4α-UMSC decreased the LPS-induced M1 polarization and enhanced M2 polarization of macrophages in Raw264.7 cells in vitro (Fig. 4e, f). Taken together, the above data indicated that co-capsulation of UMSCs and hepatocytes could improve hepatocyte viability and function, reducing inflammatory response.

Of note, previous study has identified HNF4α as a key regulator of morphological and functional differentiation of hepatocytes [42]. Therefore, we overexpressed HNF4α in UMSCs to investigate the protective effects of UMSCs in ALF mice. In vitro study showed that HNF4α-UMSCs-HEP remarkedly enhanced the viability and function of hepatocytes than UMSCs-HEP (Fig. 1d, e). In vivo, HNF4α-UMSCs-HEP could augment the therapeutic effects in ALF mice evidenced by a decreased mortality rate, reduced hepatic injury and inflammatory response compared with UMSCs-HEP group (Figs. 2, 3, and 4). Consistent with our results, a recent study reported that MSC overexpressed HNF4α could enhance the therapeutic effects on CCl4-induced mouse liver cirrhosis model by reducing inflammation and liver injury [43]. Though sharing similarities with this research, Ye’s study remain limited and differed from ours. In this research, we specifically focus on combined therapeutic effect of microencapsulation of hepatocytes and HNF4α-UMSCs in ALF mice instead of solo effect of gene-edited MSCs on chronic liver injury. Also, utilization of human umbilical MSCs qualified this research with more potential in practical translation and less ethical issue compared to bone marrow MSC isolated from mice. Beyond that, with microencapsulation as a novel drug delivering carrier, we chose peritoneal transplantation as treatment method rather than tail intravenous injection, in which method maximum viability of absorbed UMSCs could be achieved.

Mounting evidence showed that MSCs secretions exerted a beneficial effect by reducing the inflammatory response, promoting the survival and proliferation of injured cells, and ameliorating liver injury [21, 44–47]. In the present study, microcapsules were used as carriers to encapsulate UMSCs and hepatocytes to persistently release the secretory factors contributing to the repair of impaired liver and the reduction of inflammatory response in ALF. And more, HNF4α-UMSCs significantly augment the therapeutic effects on ALF mice (Figs. 1, 2, 3, 4, and 5). Protein chip assay showed that 38 proteins were significantly upregulated in HNF4α-UMSCs when compared with those of UMSCs. Analysis of GO categories revealed that the proteins in the CM of HNF4α-UMSCs are more closely associated with some pathways that regulate fundamental cellular processes, such as proliferation, differentiation, motility, stress response, apoptosis, and survival (Fig. 5d) [48]. Neutralization experiments showed that HB-EGF has significantly relieved hepatocyte injury, and no significant effects were observed in other groups (Fig. 5e). Additionally, in vivo, we found that HB-EGF neutralization antibody in mice could reverse the protection effects of HNF4α-UMSCs-HEP against ALF (Fig. 6a–d) and the switch of polarization of macrophages from M1 to M2 that was exerted by HNF4α-UMSC-HEP on ALF mice (Fig. 6e, f). These data suggested that HNF4α-UMSC-HEP played the therapeutic effects mainly mediated by HB-EGF. Interestingly, previous studies have shown that HB-EGF could protect intestine tissues from inflammatory damage by promoting the M2 polarization of macrophages in necrotizing enterocolitis injury. Moreover, HB-EGF combined with HGF obviously inhibited BDL-induced cholestatic liver injury by exerting acute cytoprotective effects and enhancing the anticholestatic effects and liver regeneration during the chronic phase [49]. In addition, we found that HNF4α binds to the HB-EGF promoter and directly upregulates the expression of HB-EGF (Fig. 6g–i). Taken together, these results indicated that HNF4α-UMSC-HEP ameliorated the ALF mice, mainly mediated via HB-EGF activated by HNF4α.

**Fig. 7:**
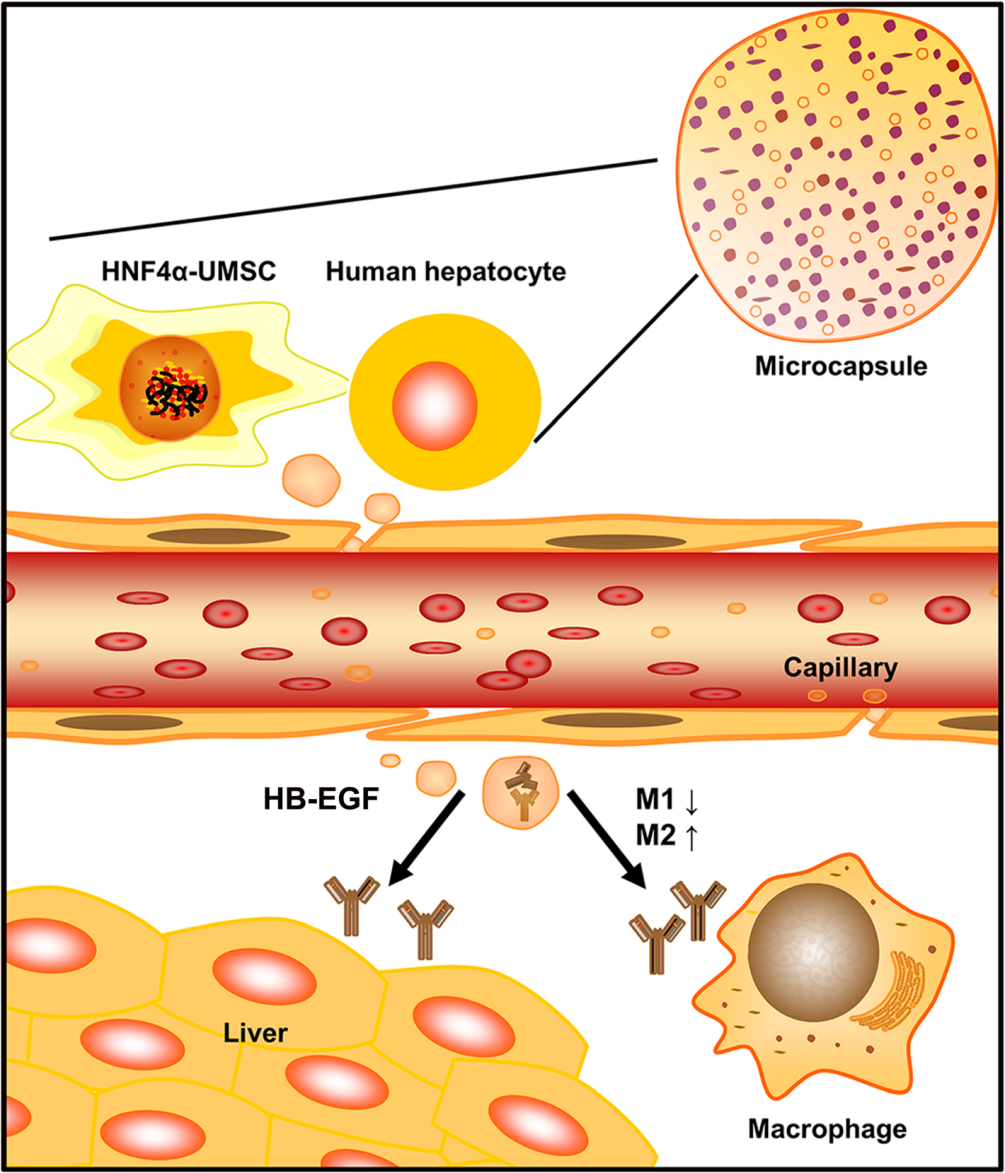
A schematic diagram illustrating the mechanisms of co-encapsulation of HNF4α-UMSCs and human hepatocytes in ALF mice injured by LPS/D-gal. “—” indicates promotion

In conclusion, the present study revealed that co-encapsulation of HNF4α-UMSCs and hepatocytes attenuated LPS/D-gal-induced liver injury and improved the survival rate of ALF mice by the reduction in the inflammatory response and promotion of macrophages polarization switch from M1 to M2, and the paracrine factors, contributing to the survival, proliferation, and metabolism of hepatocytes. In addition, HB-EGF upregulated by HNF4α plays a key role in the protective effects on ALF mice. These findings provide novel insights into the approach of cell-based therapy for ALF (Fig. 7).

## Supplementary information

**Additional file 1.** Supplementary Materials. Figure S1: Primary hepatocytes were co-encapsulated with HNF4α-UMSCs at a ratio of 10:1, 5:1, and 2.5:1. Measurement of albumin secretion and urea synthesis in the supernatant of microcapsules in different groups at varied time points. Figure S2: Assess the essential influence of HB-EGF in UMSCs on inflammation resolution effect on ALF mice. (A) Confirmation of knockdown of HB-EGF in HNF4α-UMSCs. (B) HE staining and immunochemistry images of liver sections with MPO and F4/80 antibodies (original magnification, × 200). Quantification of liver histological scores, MPO and F4/80 positive cells in sights. (C) ELISA analysis on ALT and AST concentrations in plasma of ALF mice treated with HNF4-α-UMSC-HEP and HNF4α-HFKD-UMSC-HEP. The mRNA levels of TNF-α and IL-8 levels in the liver tiusses of ALF mice treated with HNF4-α-UMSC-HEP and HNF4α-HFKD-UMSC-HEP. Table S1: The PCR primers used in the study. Table S2: The relative intensities of signals were listed in the below table and the list of relative intensities of signals of growth factors in the CMs of HNF4α-UMSCs and UMSCs which are significantly high in HNF4α-UMSCs groups. Heat map is shown in the right panel.

## Data Availability

The datasets used and/or analyzed during the current study are available from the corresponding author on reasonable request.

## Abbreviations

ALF: Acute liver failure
MSC: Mesenchymal stem cells
UMSC: Umbilical cord mesenchymal stem cells
HNF4α: Hepatocyte nuclear factor-4 alpha
APA: Alginate–poly-L-lysine–alginate
LPS: Lipopolysaccharides
D-gal: D-galactosamine
ALT: Alanine aminotransferase
AST: Aspartate transaminase
TNF-α: Tumor necrosis factor-α
IL-8: Interleukin-8
iNOS: Inducible nitric oxide synthase
PPAR-γ: Peroxisome proliferation-activated receptor-gamma
Arg-1: Arginase-1
