# Editorial Expression of Concern: Co-encapsulation of HNF4α overexpressing UMSCs and human primary hepatocytes ameliorates mouse acute liver failure

**DOI:** 10.1186/s13287-026-04962-1

**Published:** 2026-03-12

**Authors:** Defu Kong, Huiming Xu, Mo Chen, Yeping Yu, Yongbing Qian, Tian Qin, Ying Tong, Qiang Xia, Hualian Hang

**Affiliations:** 1 Department of Liver Surgery, Renji Hospital, School of Medicine, Shanghai Jiao Tong University, 160 Pujian Road, Shanghai, 200127 China; 2 State Key Laboratory of Oncogenes and Related Genes, Renji-MedX Clinical Stem Cell Research Center, Ren Ji Hospital, School of Medicine, Shanghai Jiao Tong University, Shanghai, China

**Editorial Expression of Concern: Stem Cell Research & Therapy (2020) 11:449** 10.1186/s13287-020-01962-7**IF: 7.3 Q1 B2**

The Editor-in-Chief is issuing an Editorial Expression of Concern to alert readers about concerns regarding the reporting of animal ethics approval in this article [1]. The article cites approval number SYXK 2008 0050, which was noted to appear in multiple publications describing different experiments. The authors have explained that this number refers to an Experimental Animal Use License for the animal facility rather than a study specific ethics approval and have provided documentation indicating that separate ethical approval was obtained for this study. Despite this, the reporting of animal use approval in the article and the use of a general approval instead of a specific one is inadequate. Readers are therefore advised to interpret the information regarding animal ethics approval with caution.
