# Supplementary material for: Co-encapsulation of HNF4α overexpressing UMSCs and human primary hepatocytes ameliorates mouse acute liver failure
Source: Stem Cell Res Ther. 2020 Oct 23;11:449. doi: 10.1186/s13287-020-01962-7 (PMC7583302; doi:10.1186/s13287-020-01962-7)
Supplement: Supplementary file 1 — Additional file 1. Supplementary Materials. Figure S1: Primary hepatocytes were co-encapsulated with HNF4α-UMSCs at a ratio of 10:1, 5:1, and 2.5:1. Measurement of albumin secretion and urea synthesis in the supernatant of microcapsules in different groups at varied time points. Figure S2: Assess the essential influence of HB-EGF in UMSCs on inflammation resolution effect on ALF mice. (A) Confirmation of knockdown of HB-EGF in HNF4α-UMSCs. (B) HE staining and immunochemistry images of liver sections with MPO and F4/80 antibodies (original magnification, × 200). Quantification of liver histological scores, MPO and F4/80 positive cells in sights. (C) ELISA analysis on ALT and AST concentrations in plasma of ALF mice treated with HNF4-α-UMSC-HEP and HNF4α-HFKD-UMSC-HEP. The mRNA levels of TNF-α and IL-8 levels in the liver tiusses of ALF mice treated with HNF4-α-UMSC-HEP and HNF4α-HFKD-UMSC-HEP. Table S1: The PCR primers used in the study. Table S2: The relative intensities of signals were listed in the below table and the list of relative intensities of signals of growth factors in the CMs of HNF4α-UMSCs and UMSCs which are significantly high in HNF4α-UMSCs groups. Heat map is shown in the right panel. [file 13287_2020_1962_MOESM1_ESM.docx]

**Supplementary Materials**

**Methods**

*Overexpression of HNF4α and knockdown of HB-EGF in UMSCs*

HNF4α cDNA was cloned from human umbilical cord MSCs into pHelper 1.0 plasmid. The UMSCs were then infected with lentiviral particles of HNF4Sα-GFP or GFP (used as a control). Overexpression of HNF4α was confirmed by confocal laser-scanning microscope and Western blotting. The sh-RNA for HB-EGF was constructed with reference from published literatures, then infected with HNF4α-overexpressed UMSCs. Knockdown of HB-EGF was confirmed by qRT-PCR and Western blotting

*Western blot*

Western blotting was performed to analyze the protein expression levels. The cell extracts were isolated from cultured hepatocytes using NE-PER Nuclear and Cytoplasmic Extraction Reagents (Thermo Fisher scientific, Waltham, MA, USA). The primary antibodies against HNF4α (Abcam), HB-EGF (Abcam) and β-actin as control (Sigma-Aldrich) were utilized.

*Harvest of conditioned medium*

Microcapsules of human hepatocytes, with or without HNF4α-UMSCs or UMSCs were cultured for four days, and the conditioned medium (CM) was harvested and stored at -80 °C.

*RNA extraction and real-time PCR*

RNA extraction and real-time PCR were performed as described previously.^18^ The PCR primers are listed in Table S1. Total liver RNA was extracted using TRIzol (Takara, Tokyo, Japan) reagent according to the manufacturer’s instructions. The cDNA was synthesized with PrimeScript RT reagent Kit (Takara). q-PCR was performed using CFX 96 q-PCR system (BIO-RAD, Hercules, CA, USA). A SYBR RT-PCR kit (Takara) was used for quantitative real-time PCR analysis. The relative expression levels for target gene were normalized by β-actin or GAPDH.

**Supplementary figure and table legends**

**
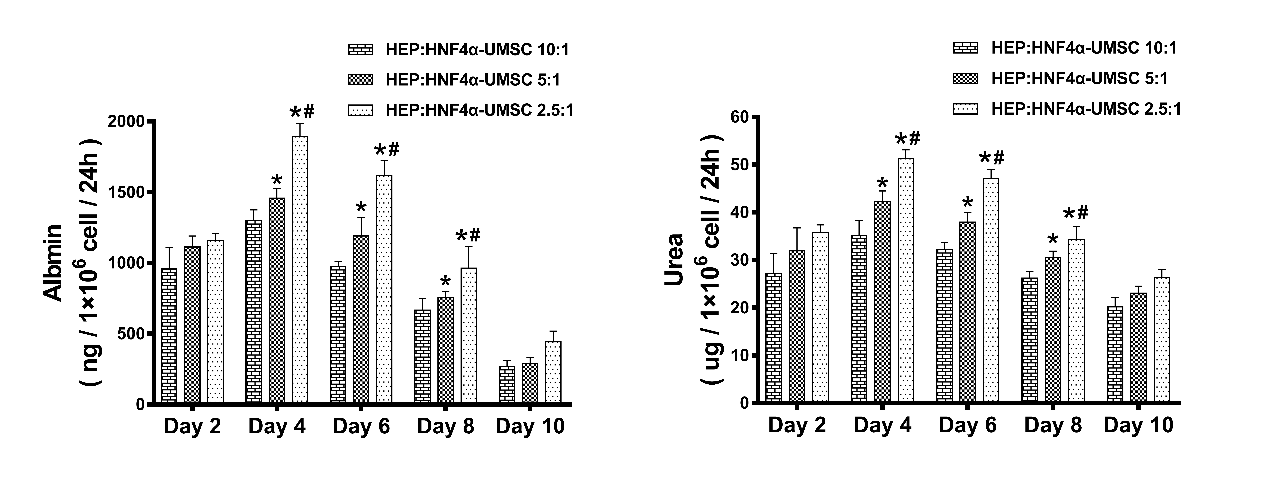
**

**Figure S1:** Primary hepatocytes were co-encapsulated with HNF4α-UMSCs at a ratio of 10:1, 5:1, and 2.5:1. Measurement of albumin secretion and urea synthesis in the supernatant of microcapsules in different groups at varied time points.


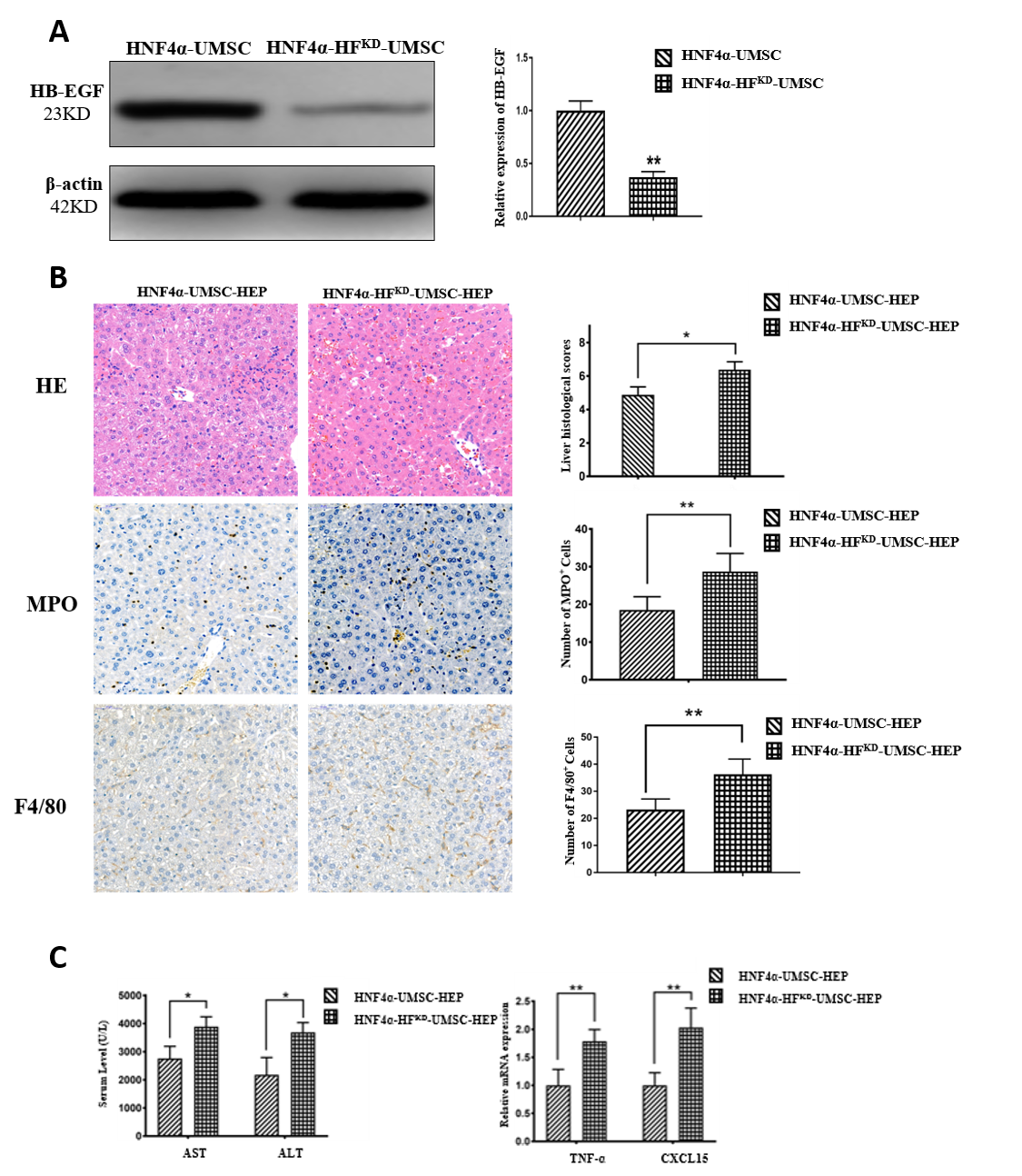


**Figure S2:** Assess the essential influence of HB-EGF in UMSCs on inflammation resolution effect on ALF mice. **(A)** Confirmation of knockdown of HB-EGF in HNF4α-UMSCs. **(B)** HE staining and immunochemistry images of liver sections with MPO and F4/80 antibodies (original magnification, × 200). Quantification of liver histological scores, MPO and F4/80 positive cells in sights. **(C)** ELISA analysis on ALT and AST concentrations in plasma of ALF mice treated with HNF4-α-UMSC-HEP and HNF4α-HFKD-UMSC-HEP. The mRNA levels of TNF-α and IL-8 levels in the liver tiusses of ALF mice treated with HNF4-α-UMSC-HEP and HNF4α-HFKD-UMSC-HEP.

| Supplementary Table 1 | | |
| --- | --- | --- |
| Gene | Forward Primer | Reverse Primer |
| Human ALB | TGCAACTCTTCGTGAAACCTATG | ACATCAACCTCTGGTCTCACC |
| Human CK18 | GGCATCCAGAACGAGAAGGAG | ATTGTCCACAGTATTTGCGAAGA |
| Human CYP3A4 | AAGTCGCCTCGAAGATACACA | AAGGAGAGAACACTGCTCGTG |
| Human GAPDH | GGCTGTTGTCATACTTCTCATGG | GGAGCGAGATCCCTCCAAAAT |
| Mouse INOS | GTTCTCAGCCCAACAATACAAGA | GTGGACGGGTCGATGTCAC |
| Mouse CD86 | TCAATGGGACTGCATATCTGCC | GCCAAAATACTACCAGCTCACT |
| Mouse TNFα | CAGGCGGTGCCTATGTCTC | CGATCACCCCGAAGTTCAGTAG |
| Mouse CXCL 15 | TCGAGACCATTTACTGCAACAG | CATTGCCGGTGGAAATTCCTT |
| Mouse Arg-1 | CTCCAAGCCAAAGTCCTTAGAG | GGAGCTGTCATTAGGGACATCA |
| Mouse Ppar-γ | GGAAGACCACTCGCATTCCTT | GTAATCAGCAACCATTGGGTCA |
| Mouse CD206 | CTCTGTTCAGCTATTGGACGC | TGGCACTCCCAAACATAATTTGA |
| Mouse β-actin | GTGACGTTGACATCCGTAAAGA | GCCGGACTCATCGTACTCC |

**Table S1**: The PCR primers used in the study.

Supplementary Table 2


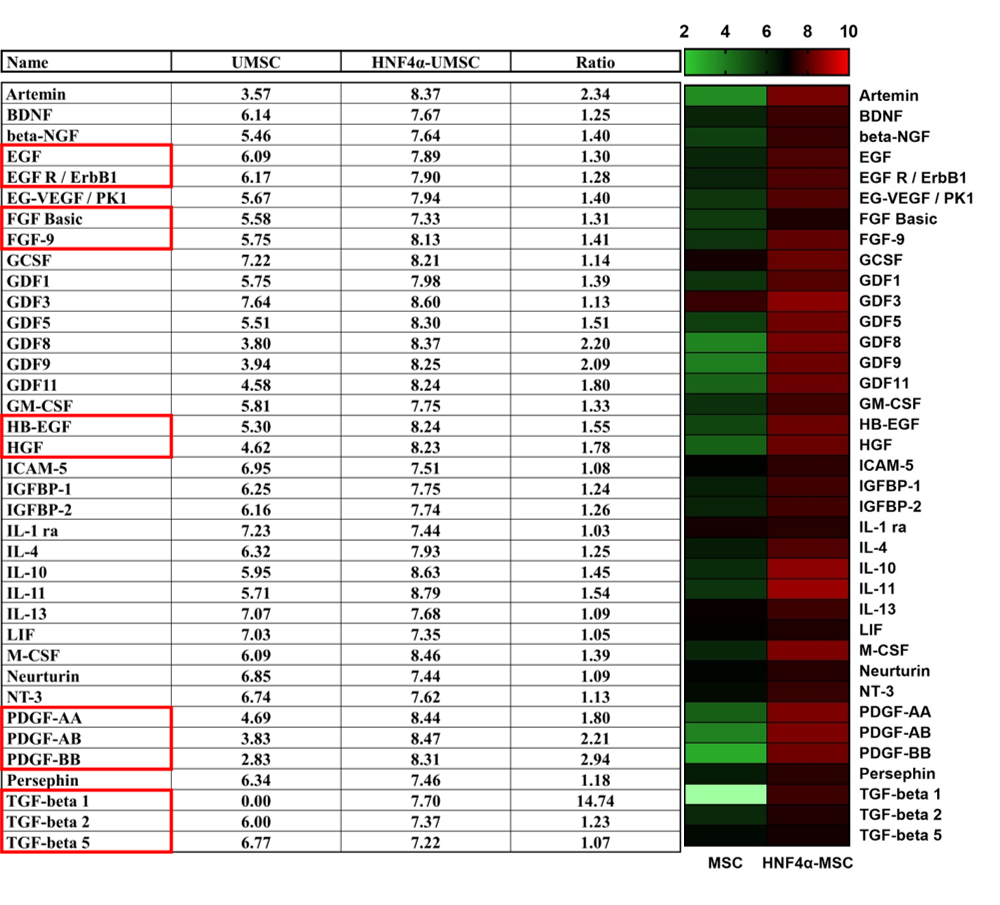


**Table S2**: The relative intensities of signals were listed in the below table and the list of relative intensities of signals of growth factors in the CMs of HNF4α-UMSCs and UMSCs which are significantly high in HNF4α-UMSCs groups. Heat map is shown in the right panel.
